# Airway Mycosis and the Regulation of Type 2 Immunity

**DOI:** 10.3390/jof6020074

**Published:** 2020-05-29

**Authors:** John Morgan Knight, Yifan Wu, Kelsey Mauk, Jill Weatherhead, Sara Anvari, Farrah Kheradmand, David B. Corry

**Affiliations:** 1 Department of Pathology and Immunology, Baylor College of Medicine, Houston, TX 77030, USA; jmk@bcm.edu (J.M.K.); Yifan.wu@bcm.edu (Y.W.); Kelsey.Mauk@bcm.edu (K.M.); farrahk@bcm.edu (F.K.); 2 Michael E. DeBakey VA Center for Translational Research on Inflammatory Diseases, Baylor College of Medicine, Houston, TX 77030, USA; 3 Department of Pediatrics, Baylor College of Medicine, Houston, TX 77030, USA; weatherh@bcm.edu (J.W.); sara.anvari@bcm.edu (S.A.); 4 Department of Medicine, Baylor College of Medicine, Houston, TX 77030, USA; 5 Biology of Inflammation Center, Baylor College of Medicine, Houston, TX 77030, USA

**Keywords:** fungi, asthma, proteinase, chronic rhinosinusitis, allergic fungal rhinosinusitis, fibrinogen, type 2 immunity, Toll like receptor 4, Th2, Th17

## Abstract

Filamentous fungi of the Aspergillus genus and others have long been linked to the induction of type 2 immunity that underlies IgE-mediated hypersensitivity responses. This unique immune response is characterized by the production of the allergy-associated T helper cell type 2 (Th2) and Th17 cytokines interleukin 4 (IL-4), IL-13, and IL-17 that drive IgE, eosinophilia, airway hyperresponsiveness and other manifestations of asthma. Proteinases secreted by filamentous fungi promote type 2 immunity, but the mechanism by which this occurs has long remained obscure. Through detailed biochemical analysis of household dust, microbiological dissection of human airway secretions, and extensive modeling in mice, our laboratory has assembled a detailed mechanistic description of how type 2 immunity evolves after exposure to fungi. In this review we summarize three key discoveries: (1) fungal proteinases drive the type 2 immune response; (2) the relationship between fungi, proteinases, and type 2 immunity is explained by airway mycosis, a form of non-invasive fungal infection of the airway lumen; and (3) the innate component of proteinase-driven type 2 immunity is mediated by cleavage of the clotting protein fibrinogen. Despite these advances, additional work is required to understand how Th2 and Th17 responses evolve and the role that non-filamentous fungi potentially play in allergic diseases.

## 1. Introduction

Filamentous fungi, especially *Aspergillus* spp., are most often acquired by inhalation of conidia. Consequently, the most common aspergillus-related diseases affect the upper and lower airways. Although the majority of research in aspergillus-related disease focuses on the highly lethal invasive syndromes involving dissemination of the fungus to other organs, the vast majority of aspergillus-related disease is non-invasive, with the organism remaining confined to the epithelial surface. Superficial airway epithelial fungal infection, termed airway mycosis, is now recognized as the fundamental cause of some of the most common of human diseases, including severe asthma, chronic rhinosinusitis, and their more severe brethren, allergic bronchopulmonary aspergillosis and allergic fungal rhinosinusitis. In addition to their localized, non-invasive nature, these syndromes are distinguished from other fungal diseases by their unique immune character, marked by the presence of eosinophils, T helper type 2 (Th2) cells, Th17 cells, and other related cell types. Collectively termed type 2 immunity, a major task of immunologists and clinicians is to understand the origins and functions of type 2 immunity and the relevance of these concepts to disease expression and management. In this review, we discuss recent research that illustrates how fungi such as *Aspergillus niger* initiate type 2 immunity and the importance of this immune response to disease expression.

## 2. Spectrum of Allergic Airway Disease

The allergic airway diseases comprise some of the most common and debilitating of all human afflictions, including asthma, chronic rhinosinusitis (CRS), and their more serious, but less common counterparts, allergic bronchopulmonary aspergillosis (ABPA) and allergic fungal rhinosinusitis (AFRS) [1]. These allergic syndromes are medically important not just for their collective mortality, estimated at 3500–4000 deaths per year in the United States for asthma alone, but also their profound morbidity, resulting in chronic disability, loss of work productivity, and loss of school time that reflect a total cost to society of more than $140 billion annually [2]. Allergic rhinitis, the least mortal of these disorders, is also the most common, affecting up to 19% of the US population especially during peak pollen seasons, adding substantially to overall morbidity. The allergic airway diseases affect children and adults and the very young and very old with similar efficiency, making these disorders a constant health threat at all life stages [1].

In addition to their consistent involvement of the airway, the allergic airway diseases are marked by a specific pattern of inflammation that includes the presence of granulocytes, most often eosinophils, but in some subjects neutrophils can be predominant; T helper type 2 (Th2) cells that secrete the cytokines interleukin 4 (IL-4), IL-5, IL-9, IL-13 and others; and of course IgE produced by B cells under the influence of IL-4. For more than 30 years, the fundamental mechanism by which the effector immune cells and molecules mediated disease expression, especially the symptoms of allergic rhinitis (rhinorrhea, nasal congestion, sneezing, facial pruritus), but also asthma (shortness of breath, cough and mucus production) was believed to be type I immediate hypersensitivity. According to this paradigm, antigen-specific IgE is bound to tissue mast cells and other cells in proximity to the airway epithelium via the high affinity IgE receptor FcεRI. Subsequent encounters with cognate antigen then crosslinks bound IgE, leading to the activation and release of diverse inflammatory mediators including histamine, proteinases, leukotrienes, and prostaglandins that coordinately promote disease expression [3].

While still helpful in explaining especially upper airway allergic diseases such as allergic rhinitis and devastating allergic disorders not primarily involving the airways such as anaphylaxis, subsequent studies have demonstrated that a fundamentally distinct immune mechanism explains the pathogenesis of especially lower airway disorders such as asthma. Type 4 hypersensitivity, in which immune effector cells more directly produce disease without going through antibody intermediates, was discovered to be the main mechanism leading to experimental asthma. The Th2 cytokine IL-13 was originally demonstrated in a mouse model of allergic airway disease to mediate airway hyperresponsiveness, a principal physiological change of the asthmatic airway in which diverse agonists provoke excessive bronchoconstriction and shortness of breath [4,5]. Subsequent work has confirmed that blockade of the shared IL-4 and IL-13 receptor subunit IL-4Rα is highly effective in relieving the symptoms of asthma [6].

Additional studies have since shown that the immune basis of asthma and related disorders includes additional, more recently described cytokines and inflammatory cells, including Th17 cells that secrete IL-17A and IL-17F; innate lymphoid cells (ILC) type 2 and 3; and the epithelial cytokines IL-25, IL-33, and thymic stromal lymphopoietin. Understanding how each component of this increasingly complex inflammatory response, collectively termed the type 2 response given the dominant role played by the Th2 cell, remains an important and highly active area of research [3]. A more fundamental question that our laboratory has been pursuing, however, is the nature of the environmental challenge that triggers and perpetuates the type 2 immune response that leads to disease chronicity.

## 3. Proteinases and Their Sources

Beginning with observations made more than a half century ago, abundant evidence eventually accumulated to implicate environmental, but especially fungal, proteinases as major drivers of allergic responses. Initial epidemiological observations revealed that the exposure to proteinases in a variety of industrial contexts invariably led to the development of occupational asthma that was often severe and difficult to manage if the subjects were not removed from the offending environments [7,8,9,10]. At the same time, allergists and other biochemically-inclined scientists began to unravel the molecular nature of allergens, that subset of antigens that drive allergic (i.e., Th2-driven) responses. They discovered that although allergens exhibit a range of biochemical functions, a surprisingly large number of allergens were either proteinases or tightly associated with proteinases [11,12]. Subsequent molecular immunological investigations revealed that key allergic airway disease-related cytokines, firstly including IL-25 [13] and subsequently IL-33 [14], are proteolytically activated, either by endogenous or dust mite proteinases.

Formal proof of the allergenic nature of proteinases eventually came through a series of experiments involving adjuvant-free proteinases derived from fungi, viruses, dust mites, cockroach, and plants such as ragweed and papaya fruit [15,16,17,18,19,20,21,22,23,24,25,26]. Among my laboratory’s findings, these studies demonstrated that diverse proteinases were highly allergenic, inducing robust allergic airway disease together with marked induction of IgE, but that proteinase activity was essential; catalytically inactivated proteinases had no allergenic activity [16]. These findings are further important because they provided an immunological explanation for the long-recognized association between two microencapsulated, airborne and easily inhaled sources of proteinase, pollen grains and fungal spores, to conditions such as asthma and allergic rhinitis. Curiously, however, fungal spores actually contain no proteinase activity; *Aspergillus* spp. only produce proteinases when growing in filamentous form, suggesting that a more complex relationship between fungi and allergic airway disease exists [27]. Specifically, these observations suggested that fungal proteinases are encountered in allergenic contexts through either the inhalation of hyphae that contain active proteinases or through the inhalation of fungal spores that then develop into proteinase-secreting mycelia in either the upper or lower airway. Although we believe that the airways are exquisitely sensitive to the presence of unregulated proteinase activity, it is essentially impossible to encounter natural environments containing sufficient aerosolized viable proteinase that a person living in that environment would achieve an allergenic dose. Allergic disease due to inhalation of active proteinase is essentially only seen in the artificial context of factories producing proteinase on industrial scales.

## 4. Fungal Infections in Allergic Airway Disease

The discovery that diverse proteinases were highly allergenic helped to explain a long-standing, but mysterious, fact of human allergic airway disease, namely that surprisingly few “allergenic” organisms are linked to disease expression, e.g., fungi, certain plants (e.g., ragweed and others), and a few arthropods found commonly in households (dust mites, cockroaches). Fungi are “professional” secretors of highly allergenic proteinases and all pollens contain abundant proteinases, which are required to complete plant reproduction. However, although dust mites and cockroaches produce proteinases, all animals—in fact—make proteinases. What was so special about mites and cockroaches, not to mention dog and cat danders, that are also conspicuously linked to human allergic diseases?

To begin to address this important question, we analyzed the dust from over 200 homes to determine the types of active proteinases could be found and if so, what organisms they derived from. We found that the dust from virtually all houses contained active proteinases, but in almost all cases the proteinases appeared to be coming from fungi, especially *Aspergillus* spp. Surprisingly, dust mite proteinases were often highly abundant in the same dust samples, but they showed no activity-they had lost their activity for reasons that remain unclear, but point to the fact that many proteinases are not designed to survive extracellularly [28].

The exception to this is fungal proteinases, which have specifically evolved to act as the extracellular digestive enzymes of fungi, breaking down organic matter so that the host organism can absorb short peptides and amino acids. We reasoned that in many households, fungal proteinases could be driving the propensity to develop allergic sensitization, potentially to themselves, but also to heterologous antigens present. In the complex antigenic milieu of household dust, we believe that the agents most likely to be recognized immunologically are simply those that are most abundant, and for many households, these most abundant antigens include non-proteolytically active moieties derived from dust mites, cockroaches, and pet danders. Despite their abundance, these antigens are unlikely to induce the production of specific IgE responses themselves, but rather are more likely to drive tolerogenic responses. However, if these common antigens are first encountered in the context of active proteinases, the more typical tolerogenic response switches to an effector Th2 and IgE-predominant response. Our research in fact demonstrated that fungal proteinase inhaled by mice at the same time as the otherwise innocuous tolerance-inducing antigen ovalbumin drives heterologous ovalbumin-specific Th2 and IgE responses [16].

The absolute amount of fungal proteinase activity present in the average household was very small however, almost certainly insufficient to induce allergic sensitization as compared to the relatively high concentrations of proteinase inhaled in industrial contexts that are strongly associated with asthma [7,8,9,29,30]. These observations collectively suggested the existence of a more complex relationship between fungi, fungal proteinases, and human allergic airway disease. Of all the household organisms linked to allergic airway disease, only the fungi are capable of infecting the human upper and lower airways, persisting for long periods and growing and reproducing over time as has been observed in allergic disorders such as allergic bronchopulmonary aspergillosis [31,32]. However, can fungi be found routinely in the airways of the more common allergic disorders asthma and CRS?

A standard answer to this question is “no”, and for good reason: respiratory mucus cultures in the context of asthma and CRS are routinely negative, that is they only uncommonly yield growth of either bacteria or fungi. This highly predictable outcome was perplexing to us, because careful microscopic examination of human respiratory tract mucus from subjects suffering from severe asthma or CRS essentially always revealed fungal elements including yeast cells, spores, and hyphae, suggesting robust colonization or active infection [33,34]. To resolve the paradoxical culture results of respiratory tract mucus, we reasoned that anti-fungal elements, including antimicrobial peptides, present in mucus were likely suppressing the growth of fungi (and other organisms); most microbiology laboratories do not attempt to separate culturable organisms from the mucus. We therefore developed an improved culture method in which airway mucus was liquefied by the addition of a reducing agent (dithiothreitol). We then performed a centrifugation step to remove culturable organisms from the mucus, allowing us to distribute on microbiological media the organisms free from possible growth-suppressing elements [33].

The results of this new culture method were striking. For our initial application to five patients with status asthmaticus receiving mechanical ventilation for respiratory failure, our laboratory identified multiple mold species from bronchoalveolar lavage specimens that the simultaneously determined hospital microbiology laboratory failed to reveal. A notable exception was the hearty yeast *Candida albicans*, which grew regardless of the culture method. We have since applied this same method to the analysis of almost 1000 spontaneous human sputum samples obtained from United States Veterans at the Michael E. DeBakey VA Medical Center. This new method has yielded one or more fungus in 87% of patients with asthma [35]. Based on results from a smaller sample size, we estimate that the fungal yield from the culture of similar specimens using standard technique is approximately 5–10%.

We further extended this method to the analysis of maxillary sinus lavage fluid from patients undergoing endoscopic sinus surgery for a variety of indications [34]. We found that the yield of any fungus from the culture of this material in the context of allergic disease (allergic or eosinophilic CRS with and without asthma) was approximately 75%, whereas the fungal yield from similar specimens obtained from patients without allergic disease was approximately 18%. We furthermore determined the frequency of fungal sensitization in these patient groups, assessing whether patients harbored fungus-specific Th2 cells within their peripheral blood mononuclear cells. Using a panel of fungal antigens derived from the nine most common fungi isolated from respiratory tract cultures, we found that almost 70% of patients with allergic disease reacted positively to one or more fungi in the panel, whereas none of the control patients reacted. However, the fraction of responders jumped to 89% if the fungal antigen used for T cell restimulation was matched to a fungus actually cultured from the same patient, whereas again the control group showed no reactivity [34].

These extensive series of studies demonstrate unequivocally that the majority of subjects with severe forms of asthma and CRS have underlying airway fungal infections. We term this new form of chronic, non-invasive airway fungal infection *airway mycosis*. Nonetheless, many clinicians still question whether the fungi are etiologic with respect to disease expression. To address this further, we challenged mice with the spores of many of the fungi we have cultured from human respiratory tract mucus, finding in every case that fungi, including *C. albicans*, can grow within the normal mouse airway and produce allergic lower airway disease that is in many respects identical to the human counterpart, including the development of airway hyperresponsiveness [27,33,36]. Moreover, we demonstrated that filamentous fungi rendered genetically incapable of secreting normal amounts of proteinases are incapable of inducing asthma-like disease in mice, confirming the ultimate importance of secreted fungal proteinases as essential virulence factors for the induction of type 2 responses and allergic airway disease [27]. Consequently, multiple published clinical trials and retrospective clinical analyses have demonstrated the benefits of treating asthma with antifungal agents [35,37,38]. Nonetheless, these findings do not rule out the possibility that non-infectious fungal hyphae can also be inhaled and mediate type 2 immunity through the active proteinases they contain.

## 5. Proteolytic Induction of Type 2 Immunity

The overwhelming importance of fungi and their proteinases to allergic airway disease motivated us to explore the molecular mechanisms by which filamentous fungi elicit allergic airway disease. Previous work had suggested that the lipopolysaccharide (LPS) receptor Toll like receptor 4 (TLR4) was important for the expression of mouse allergic airway disease induced by house dust mite allergy in part due to a structural resemblance between the TLR4 co-receptor MD-2 and the major dust mite allergen Der p 2 [39,40,41]. We initially determined the importance of TLR4 for expression of mouse allergic airway disease induced by the fungal proteinase PAO (proteinase of *Aspergillus oryzae*), but extended this work to allergic disease induced by ovalbumin and viable *A. niger* spores. We found in all three contexts that TLR4 was required for robust expression of eosinophilic inflammation and airway hyperresponsiveness, but interestingly was not required for Th2 and IgE responses [25], indicating that in these contexts, TLR4 was essential to the induction of the innate component of the global type 2 immune response, but not the adaptive side controlled by Th2, Th17 and B cells.

Having found a key innate immune receptor responsive to allergenic proteinases, our next task was to discover the putative ligand, presumably one deriving proteolytically from an endogenous substrate. We immediately focused our attention on fibrinogen for the following reasons: (1) the massive deposition of fibrin in the asthmatic airway (plastic bronchitis) demonstrates that fibrinogen is being cleaved in the airway, potentially generating immunologically-active fragments [42]; (2) fibrinogen is known to interact as a ligand with TLR4 [43]; (3) fibrinogen is the functional equivalent of coagulogen and pro-spatzle, arthropod proteins that are proteolytically cleaved to yield peptides that bind to arthropod Toll, the functional equivalent of TLR4, to mediate antifungal immunity [44]. We therefore subjected human fibrinogen to cleavage by PAO and a closely related proteinase, PAM (proteinase of *Aspergillus melleus*), finding that indeed these proteinases cleave whole fibrinogen into a variety of fibrinogen cleavage products (FCPs), especially in the 75–150 kD size range [45].

To determine if these fragments are biologically active in ways that are relevant to the expression of allergic airway disease, we tested them in two assays, adding FCPs to (1) cultures of bone marrow derived macrophages to assess their ability to enhance innate antifungal immunity and (2) giving FCPs intranasally to naïve mice to determine if they induce innate allergic airway disease. We discovered that macrophages are activated by FCPs to enhance production of diverse antifungal proteins and that their ability to inhibit the growth of filamentous fungi in vitro was strongly enhanced. Moreover, we confirmed that this FCP-driven fungistatic activity was induced entirely through TLR4. We further demonstrated that FCPs given to mice induce allergic airway disease, including airway hyperresponsiveness, again through TLR4 [25,45].

**Figure 1:**
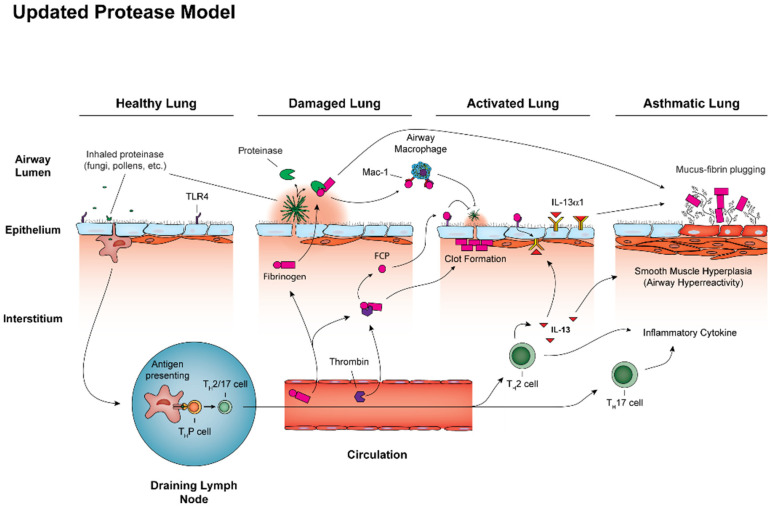
Airway mycosis coordinates activation of the airway type 2 immune response in allergic airway disease. All persons are exposed to inhaled irritants such as pollens, but also infectious agents such as fungal spores. These exposures may trigger innate and adaptive immune responses, including Th2 and Th17 cell activation, through antigen presentation by dendritic cells, but usually without inducing disease. Lungs can become damaged, however, should fungi gain a foothold within the airway lumen and begin producing proteinases. These enzymes cleave fibrinogen into fibrinogen cleavage products (FCP) that bind to the integrin Mac-1 and ultimately Toll like receptor 4 (TLR4) to activate innate antifungal immunity through macrophages and epithelial cells. With more advanced degrees of airway mycosis, potentially due to a failure of antifungal mechanisms, additional changes can occur to activate the lung, including FCP-dependent induction of chemokines to promote recruitment of allergic effector cells (e.g., Th2 cells, Th17 cells, eosinophils) to the airways and also upregulation of IL-13Rα1, the main binding chain of the IL-13 receptor. Allergic airway disease results from sustained lung activation due to fungal proteinases, resulting in attendant remodeling that includes goblet cell metaplasia with mucus hypersecretion, smooth muscle hypertrophy resulting in airway hyperresponsiveness, and overall greater degrees of allergic inflammation and airway clot formation (plastic bronchitis).

An additional discovery from these studies was that FCPs, derived largely from the fibrinogen D domain [45], do not bind to TLR4 directly, but rather indirectly by first binding to the integrin CD11b/CD18 (Mac-1). Thus, both mice deficient in Mac-1 and mice expressing a mutant fibrinogen that is unable to bind to Mac-1 [46] failed to develop robust allergic airway disease in response to fungal challenge [45]. Together, these findings suggest a molecular mechanism by which fungal proteinases cleave airway fibrinogen into FCPs that then bind to Mac-1 to then engage TLR4 to mediate both innate fungistatic immunity and airway type 2 immune responses. Because type 2 immunity is, in aggregate, a complex antifungal immune response [36], the FCP-Mac-1-TLR4 axis is therefore an extremely important antifungal effector pathway incorporating danger signals from a pathogen (fungal proteinase) with ancient components of the mammalian immune early warning system that includes TLR4 and fibrinogen (Figure 1). In support of these findings, interruption of the clotting system by administering anticoagulants such as heparin has previously been shown to be beneficial in asthma [47,48]. Additionally, TLR4 impacts the expression of asthma in complex ways, such that multiple TLR4 ligands have been shown to enhance disease expression in mice [25,39,49] and TLR4 polymorphisms confer either enhanced [50] or reduced [51] asthma susceptibility.

## 6. Concluding Remarks

Despite the explanatory power of the newly discovered FCP-TLR4 pathway in defining the molecular basis of type 2 immunity and allergic airway disease, we still do not know how Th2 and Th17 cells arise in the context of airway mycosis, although we do know that fungal proteinases strongly drive these responses. In future studies, our laboratory will explore the Aspergillus proteinase pathways that lead to Th2 and Th17 development, considering also whether yeast pathogens such as *Candida albicans* induce type 2 immunity through similar mechanisms. We commonly isolate *C. albicans* from human sputa as well as bronchoalveolar lavage [33,35] and this organism induces mouse allergic airway disease and type 2 immunity to the same degree as filamentous fungi [33]. As we begin to consider novel therapeutic strategies to counter proteinase and fibrinogen-dependent allergic inflammation, it will be critical to understand if the molecular pathways described here are applicable to all fungi or only a subset. It will further be important to determine if defects in this pathway explain, in part, the chronicity of airway mycosis that underlies the otherwise unexplained persistence of allergic airway diseases in those with apparently normal immune responses.
